# Correction to “Preoperative Gemcitabine/Nab‐Paclitaxel With Concurrent Radiation Therapy for Borderline Resectable Pancreatic Cancer: A Comparative Analysis With Preoperative Chemoradiation Therapy Using Gemcitabine Alone”

**DOI:** 10.1002/ags3.70277

**Published:** 2026-09-03

H. Akita , H. Takahashi , Y. Mukai , et al., “Preoperative Gemcitabine/Nab‐Paclitaxel With Concurrent Radiation Therapy for Borderline Resectable Pancreatic Cancer: A Comparative Analysis With Preoperative Chemoradiation Therapy Using Gemcitabine Alone,” Annals of Gastroenterological Surgery 10, no. 4 (2026): 1209–1216, 10.1002/ags3.70191 42395141 PMC13327093.

In the published article, the affiliation was incorrectly listed as “Department of Gastroenterological Surgery, The University of Osaka, Suita City, Osaka, Japan.” The correct affiliation is “Department of Gastroenterological Surgery, Osaka International Cancer Institute, Osaka, Japan.”

The online article has been corrected.

We apologize for this error.

